# Supplementary material for: Health and Wellness Projects Created by Student Pharmacists during Advanced Pharmacy Practice Experiences: Exploring the Impact on Professional Development
Source: Pharmacy (Basel). 2023 Dec 28;12(1):5. doi: 10.3390/pharmacy12010005 (PMC10801478; doi:10.3390/pharmacy12010005)
Supplement: Supplementary file 1 [file pharmacy-12-00005-s001.zip › Document S1 FAQs for Preceptors.pdf]

Information of Preceptors

Frequently Asked Questions Information for the Student-created and implemented Community APPE Health & Wellness Project

- What are my responsibilities as preceptor? *The student must discuss all ideas with the preceptor and get his/her approval of the project. The student is responsible for carrying it out and the preceptor is only to act as a supervisor/mentor.*
- What about any costs incurred (for materials, copying, poster board, etc.)? *The voucher money may be used for that. Submit the bill to Experiential Education. Personnel hours other than the student are not permitted as the student will be conducting the project.*
- Can the project be held outside of the student's regularly scheduled hours? *Yes, any variation of the hours should be reported on the certification of hours form.*
- Can this be part of the site's regular outreach program? *Yes, but the student must take charge of that day's entire program, developing educational materials, setting up the area, interacting with the patients, keeping track of the number of community participants, and developing and conducting a patient satisfaction survey*
- Who will contact the corporate office (if in a chain)? *The student will work with the preceptor, pharmacy manager (if not the preceptor), front store manager and district manager to gain acceptance of the project*
- What happens if no one comes and/or there is no patient interest or involvement? *In the unfortunate event that no one participates (or there is very poor participation), the student will prepare a "post-project" assessment listing possible reasons for lack of participation and outline possible strategies for improving participation.*
