# Supplementary material for: Health and Wellness Projects Created by Student Pharmacists during Advanced Pharmacy Practice Experiences: Exploring the Impact on Professional Development
Source: Pharmacy (Basel). 2023 Dec 28;12(1):5. doi: 10.3390/pharmacy12010005 (PMC10801478; doi:10.3390/pharmacy12010005)
Supplement: Supplementary file 1 [file pharmacy-12-00005-s001.zip › Table S1 Health and Wellness Project Grading Rubric.pdf]

Table S1. Health & Wellness Projects will be evaluated by Experiential Education using the following Rubric:

Project elements	2= Exemplary	1= Acceptable	0= Unacceptable
Description of project	• Project objectives are stated clearly, **and** in a way that demonstrates student understanding of the health and wellness needs 2.3, 2.4<br>• Project intervention addresses identification, prevention and/or management, for individuals or specific populations to improve health and wellness 2.3,2.4 3.3 **and**<br>• Project evaluates personal, social, economic **and** environmental conditions to maximize health and wellness 3.4 **and**<br>• Project is a creative and/or unique way to address the health and wellness objective4.3<br>• Creates a long-term or ongoing project over the course of the entire rotation	• Briefly lists objectives pertinent to the project<br>• Project addresses one or more of the following for individuals or specific populations to improve health and wellness:<br>○ management<br>○ prevention<br>○ intervention<br>• Project evaluates one or more of the following:<br>○ personal<br>○ social<br>○ economic<br>○ environmental conditions to maximize health and wellness<br>• Scope of the project is appropriate<br>• In an already established program provides a unique perspective/input	• Project does not address management **or** prevention **or** intervention **or** educational strategies for individuals or specific populations to improve health and wellness<br>• Project does not evaluate personal **or** social **or** economic **or** environmental conditions to maximize health and wellness.<br>• Does not list objective of the project<br>• Participates in an already established program with no student input (other than attendance)
Materials	• Creates original materials specific to the project 4.3<br>• Provides a wide variety of materials, in more than one format<br>• Language and information is tailored for intended population3.5.3, 3.2, 3.6 at appropriate literacy level, language.	• Provides informational materials<br>• Uses pre-printed, non-original materials<br>• Minimal spelling and grammar mistakes<br>• Language is clear and patient appropriate	• No materials uploaded or described<br>• Many spelling and grammar mistakes<br>• Unprofessional language is used often (texting language)

	• Materials are professional-looking, neat, visually appealing. Patient friendly language used. Information is accurate. 2.1<br>• References are cited and/or provided	• References cited/provided	• Language is not patient friendly<br>• References not cited
Addressing the Standards	• Addresses 3 or more standards3.3, 2.1, 3.2, 3.5.3, 2.3, 2.4, 3.5<br>• Provides specifics for each standard cited **and**<br>• States how this will address management, prevention **and** intervention of the specific population **and**<br>• Defines what outcomes are to be expected	• Addresses <3 standards **OR**<br>• Provides non-specific or generic examples **OR**<br>• Project addresses one or more of the following:<br>○ management,<br>○ prevention<br>○ intervention **OR**<br>• Does not define what outcomes are to be expected	• Addresses< 2 standards **and**<br>• Provides non-specific or generic definition of the standard **and**<br>• Does not address management, prevention or intervention **and**<br>• Does not provide expected outcomes
Post project summary	• Project outcomes are described in detail<br>• Description of the factors contributing to the outcomes of the program show student reflection on the planning and implementation of the program 3.1<br>• Detailed information pertaining to the attendance and number of interventions conducted is included (specific numbers)	• Project outcomes and factors contributing to the outcomes are clearly stated<br>• Brief/generic information is given pertaining to the attendance/number of interventions conducted	• Post-project summary is missing<br>• Summary is too brief and generic to understand the outcomes
Essay	• Shows expanded thought processes in describing a pharmacist's role in health and wellness 4.1, 4.3, 4.4<br>• Exhibits self-awareness in relating health and wellness to future career goals/plans4.1	• Adequately describes a pharmacist's role in health and wellness<br>• Explains how this role will impact the student's future career	• Does not accurately describe a pharmacist's role in health and wellness<br>• Does not link to student's future career

Exemplary project = 4 out of 5 exemplary scored project elements

Unacceptable = an unacceptable score in a project element requires revision of that element
